# Head injury in older adults presenting to the ambulance service: who do we convey to the emergency department, and what clinical variables are associated with an intracranial bleed? A retrospective case–control study

**DOI:** 10.1186/s13049-023-01138-1

**Published:** 2023-10-31

**Authors:** J. W. Barrett, J. Williams, S. S. Skene, J. E. Griggs, D. Bootland, J. Leung, A. Da Costa, K. Ballantyne, R. Davies, R. M. Lyon

**Affiliations:** 1 South East Coast Ambulance Service NHS FT, Crawley, UK; 2 University of Surrey, Guildford, UK; 3 Paramedic Clinical Research Unit, University of Hatfield, Hatfield, UK; 4 Air Ambulance Charity Kent, Surrey and Sussex, Redhill, UK; 5 University Hospitals Sussex NHS Foundation Trust, Worthing, UK; 6 East Kent Hospitals University NHS Foundation Trust, Canterbury, UK; 7 Medway Maritime Hospital NHS FT, Gillingham, UK

## Abstract

**Objective:**

Most older adults with traumatic brain injuries (TBI) reach the emergency department via the ambulance service. Older adults, often with mild TBI symptoms, risk being under-triaged and facing poor outcomes. This study aimed to identify whether sufficient information is available on the scene to an ambulance clinician to identify an older adult at risk of an intracranial haemorrhage following a head injury.

**Methods:**

This was a retrospective case–control observational study involving one regional ambulance service in the UK and eight emergency departments. 3545 patients aged 60 years and over presented to one regional ambulance service with a head injury between the 1st of January 2020 and the 31st of December 2020. The primary outcome was an acute intracranial haemorrhage on head computed tomography (CT) scan in patients conveyed to the emergency department (ED). A secondary outcome was factors associated with conveyance to the ED by the ambulance clinician.

**Results:**

In 2020, 2111 patients were conveyed to the ED and 162 patients were found to have an intracranial haemorrhage on their head CT scan. Falls from more than 2 m (adjusted odds ratio (aOR) 3.45, 95% CI 1.78–6.40), chronic kidney disease (CKD) (aOR 2.80, 95% CI 1.25–5.75) and Clopidogrel (aOR 1.98, 95% CI 1.04–3.59) were associated with an intracranial haemorrhage. Conveyance to the ED was associated with patients taking anticoagulant and antiplatelet medication or a visible head injury or head injury symptoms.

**Conclusion:**

This study highlights that while most older adults with a head injury are conveyed to the ED, only a minority will have an intracranial haemorrhage following their head injury. While mechanisms of injury such as falls from more than 2 m remain a predictor, this work highlights that Clopidogrel and CKD are also associated with an increased odds of tICH in older adults following a head injury. These findings may warrant a review of current ambulance head injury guidelines.

## Introduction

Most older adults with a traumatic brain injury (TBI) will present to the emergency department (ED) via the ambulance service [1]. A core component of ambulance clinician's activity is to diagnose and triage their patients to the most appropriate care pathway. However, identifying traumatic intracranial haemorrhage (tICH) in older TBI patients is a diagnostic challenge for ambulance clinicians; a tICH cannot be diagnosed without a head computed tomography (CT) scan [2]. Therefore, ambulance clinicians must use surrogate markers to determine whether their patients, typically presenting with a closed head injury, will likely have a tICH. Traditionally, these markers have been a period of loss of consciousness, amnesia or a persistent reduction in the patient's Glasgow Coma Scale (GCS) score [3].

Older adults have gained considerable attention in trauma care research and remain a high priority; in the context of TBI, older adults typically find their care under-triaged and are more likely to suffer poor outcomes [4]. In addition, this growing patient population typically present with mild symptoms, even when they suffer significant tICH [5]. Subsequently, evidence suggests that ambulance clinicians struggle to identify older adults with a TBI [6, 7].

Although ambulance services in England report a non-conveyance rate between 40 and 68% [8], the conveyance of older adults with a head injury has been reported to be as high as 70% [9]. Current guidelines available to ambulance clinicians, such as the Joint Royal Colleges' Ambulance Liaison Committee (JRCALC) head injury guidelines, are based on studies conducted in the ED. This reflects the lack of evidence from the pre-hospital environment as head-injured older adults non-conveyed by the ambulance service are a population not routinely reported in the head injury or TBI literature.

This study aimed to determine whether there was sufficient information at the scene of injury for an ambulance clinician to identify which older adults with a head injury were at risk of tICH. A secondary aim was to describe which older adults presenting to the ambulance service with a head injury were most likely to be conveyed to the ED.

## Methods

### Study setting

South East Coast Ambulance Service NHS Foundation Trust (SECAmb) provides unscheduled urgent and emergency care in the South East of England, responding to 999 and 111 calls throughout Kent, Surrey and Sussex. Operating across a geographical region of 3000 square miles and serving a population of approximately four million people, SECAmb has access to three trauma networks (Southwest London and Surrey Trauma Network, Sussex Trauma Network, and Southeast London, Kent and Medway Trauma Network). Each network comprises one Major Trauma Centre (MTC) and several Trauma Units (TU). As well as paramedics and non-registered health care professionals, SECAmb have specialist paramedics in urgent and critical care and are supported by Air Ambulance Charity Kent, Surrey and Sussex, which provides 24/7 physician-paramedic Helicopter Emergency Medical Services (HEMS). Data for this study were collected from one major trauma centre, six trauma units and one general district hospital based in the Sussex Trauma Network and Southeast London, Kent and Medway Trauma Network.

### Patient population

Patients were considered for inclusion if they were 60 years or older, presented to a SECAmb crew in the geographical region of one of the participating hospitals, and had an acute head injury occurring within the 24 h preceding the 999 or 111 call. Patients were excluded if they were 59 years old or younger, presented with a head injury more than 24 h after the time of the 999 or 111 call, or refused an ambulance crew. In cases where patients were conveyed to the ED, they were excluded if they refused a head CT scan or left the hospital before a clinician assessed them.

### Study design and data collection

This was a retrospective observational case–control study. Patient registries of participating hospital EDs were searched for patients matching the inclusion criteria. Cases were patients found to have a tICH on their head CT scans, and controls were patients with no tICH found. Both presenting reason and ED diagnosis were searched for terms such as head injury and TBI (and related variations), as recorded by Emergency Care Data Set (ECDS) and SNOMED codes (“Appendix A”). The following information was extracted from the patient's ED record, date and time of arrival, whether the patient received a head CT scan and time of that scan, and admission status (discharged from ED, admitted to hospital).

For patients who received a head CT scan, their scan report, completed by a radiologist at the time of the scan, was reviewed by a study team member and categorised as tICH found or no tICH found. Where a tICH was reported, these were classified as Extradural (EDH), Subdural (SDH), Subarachnoid (SAH) or Other. Neurosurgical referral, acceptance or decline (with reason where available) were also collected. These ED records were then linked to their corresponding ambulance electronic patient report form (ePCR). The SECAmb Business Intelligence (BI) department linked ePCR to ED records through three phases sequentially:

1. Each SECAmb incident generates a unique eight-digit incident code, and this is passed onto the ED. Cases were matched on this primarily
2. Patients were matched according to their unique NHS number and their date of birth in the absence of an incident code-limited to the date of the patients attendance
3. If the previous two approaches were unsuccessful, the time and date of when an ambulance arrived at the hospital and the time and date of the patient being admitted into the ED were used

The clinical variables for data extraction were identified a priori and extracted from several domains in the ambulance ePCR: anonymous patient demographics (Gender, Age, Ethnicity), current anticoagulant or antiplatelet status, clinical observations and head injury-related clinical assessments (reduced GCS, loss of consciousness, focal neurological deficit, suspicion of skull fracture, headache, vomiting, post-injury seizure, amnesia), the type of head injury (i.e. bruising, laceration, skin tear, etc.) and the mechanism of injury (Falls < 2m, Falls > 2m, road traffic collision [RTC], other or unknown). A limited number of comorbidities were also selected and included Atrial Fibrillation, Hypertension, Alcohol Dependence, previous Stroke or Myocardial Infarction [MI], Ischemic Heart Disease, Heart failure, Chronic Obstructive Pulmonary Disease, Dementia, Alzheimer's Disease, Diabetes Mellitus and Chronic Kidney Disease [CKD], due to their association with TBI [10, 11] or their association with an increased risk of falls [12]. Demographic, comorbidity, prescribed medication and clinical observation data were available from designated inputs in ePCR. Clinical assessment data had to be extracted from free text boxes within the ePCR and was conducted by JWB and RD using a data collection tool.

Data from ePCRs were also collected from patients presenting to SECAmb seen in the same geographical region of the participating hospitals but were non-conveyed and recorded as having a head injury. This created a unique dataset of head-injured older adults presenting to the ambulance service, detailing their journey from time of 999/111 call to ambulance or ED discharge.

### Data handling

The predefined clinical symptoms and injuries were found to have a high degree of missingness, which resulted in their removal. To salvage missing data, composite variables were created following data collection and were a deviation of the study protocol. *Head injury present* was a variable to represent any recording of a viable injury above the clavicles instead of the itemised approach. *Head injury symptom* represents the presence of any symptoms related to a head injury from the predefined clinical variables.

Furthermore, the dataset contained a group of patients whose GCS was less than 15 due to a pre-existing cognitive condition. To ensure the analysis was not bias, the variable *abnormal GCS* was created; this variable acknowledges whether the GCS for the patient is abnormal for the patient. To validate this, clinical records were reviewed in cases where the patient's GCS was lower than 15, and new head injury related symptoms were considered to denote that the GCS was abnormal for the patient. If no record was made, but the patient had a pre-existing cognitive impairment, this was considered normal for the patient.

### Primary and secondary outcome

The primary outcome was a tICH found on head CT scan in patients conveyed to the ED. Secondary outcomes were factors predictive of conveyance to the ED by the ambulance service. A tICH was defined as EDH, SDH, SAH, and classified as TBI likely; patients who did not have a tICH on their CT scan or did not receive a head CT scan were classed as TBI unlikely. Including patients in this cohort who did not have a head CT scan was justified, given that those patients who refused a head CT scan or left before assessment were excluded and that the threshold for a head CT scan in older adults is typically low in EDs [13]. As such, the assumption was made that the clinician likely had a very low suspicion of a TBI.

### Statistical analysis

Patients were described by their demographic and clinical aspects according to whether they were conveyed or non-conveyed to the ED. Conveyed patients were described according to the presence of a tICH on their head CT. Descriptive statistics were used to describe the differences between groups; given the patient population being studied, continuous data were assumed to be skewed and presented as median with interquartile ranges (IQR), and categorical data were reported in frequency and proportions (%). Differences between groups were measured through inferential statistics, Mann Whitney-U test was used in continuous variables and chi-square tests for categorical data. Binary logistic regression models were created to determine which clinical predictor variables were associated with a patient having a tICH on their head CT scan. Stepwise variable selection methods were used to identify which clinical predictor variables should be used in the model. Odds ratios were extracted from the models' coefficients and adjusted for the other variables in the model. In variables with no more than 10% missingness, multiple imputations of chain equations (MICE) [14, 15] was implemented with 50 iterations. Sensitivity analysis was performed by analysing convergence plots of the imputations and whether there would be a difference from performing a complete case analysis (all missing data removed from the dataset). This analysis supported the use of the imputed data, thus, complete case analysis was discarded. Data were analysed in R (Version 4.2.1).

## Results

**Fig. 1:**
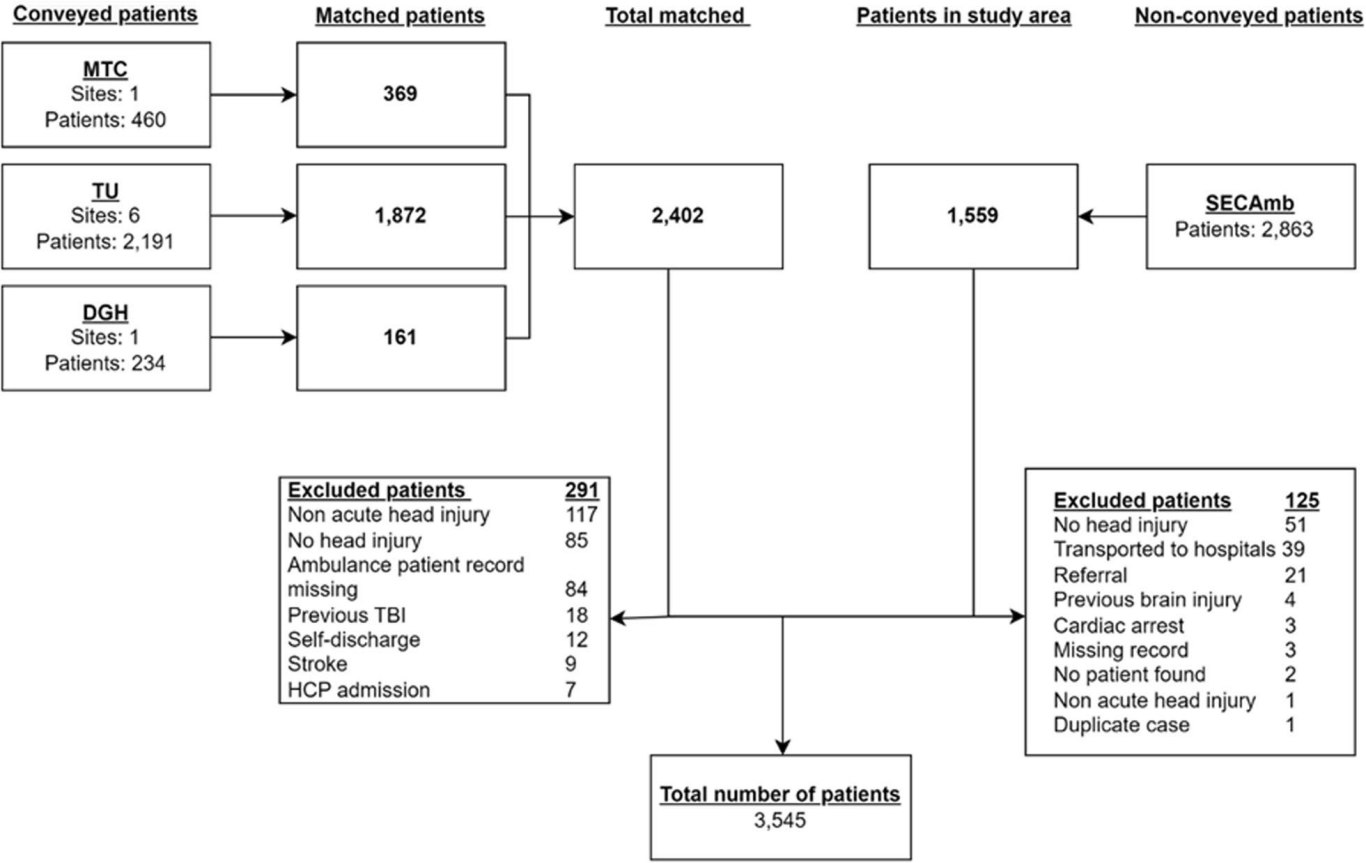
Patient flow depicting how many cases were matched and reasons for exclusion of cases. DGH, District General Hospital; HCP, Healthcare professional; MTC, Major trauma centre; TBI, Traumatic brain injury; TU, trauma unit

**Fig. 2:**
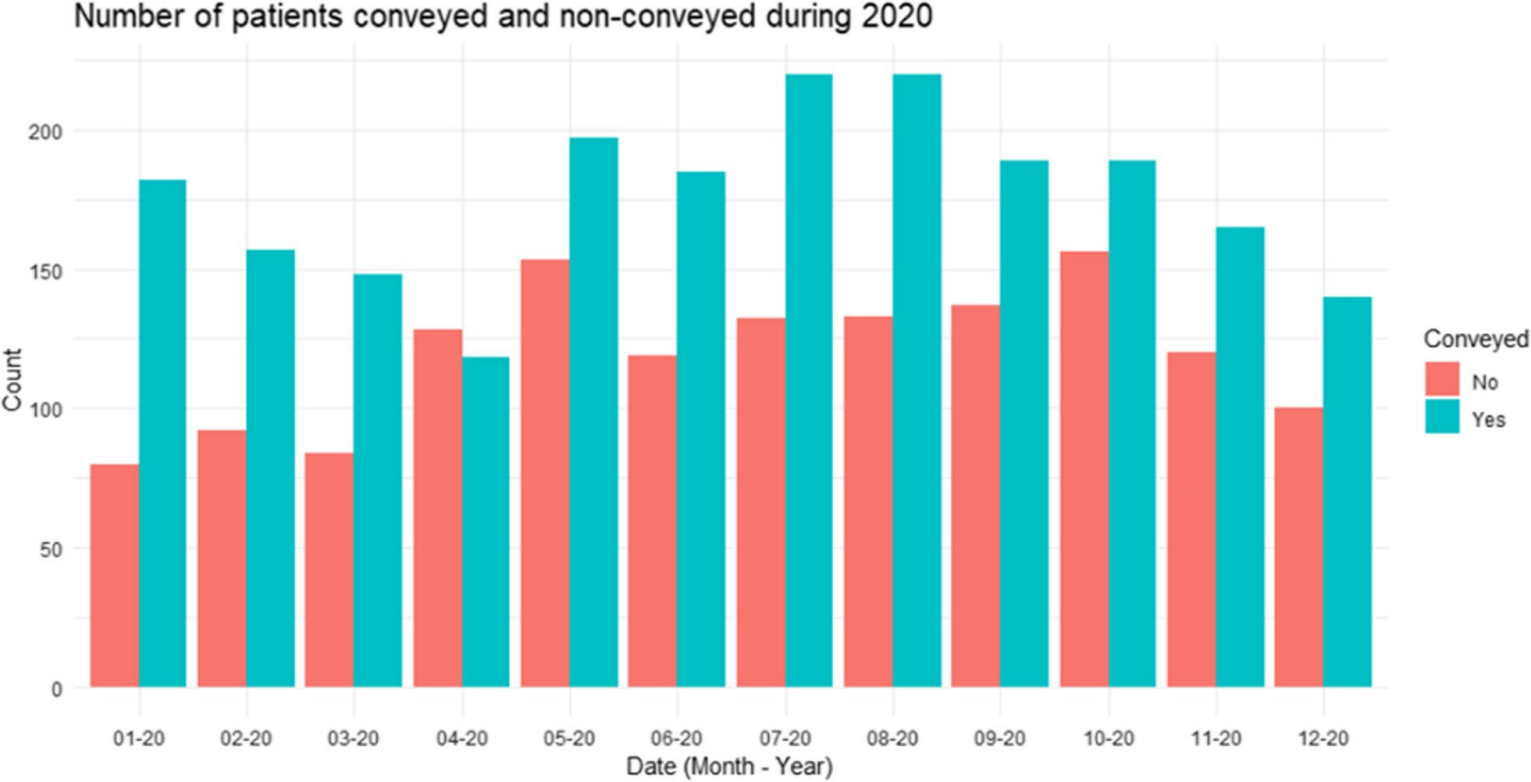
Comparison of the frequency of patients conveyed and non-conveyed during the study period

From the 1st of January 2020 to the 31st of December 2020, 2885 patients conveyed to the eight participating EDs, and 2402 patients (83.32%) cases were matched to a SECAmb ePCR. There were 291 (12.11%) patients excluded following screening; the most frequent reason for exclusion was patients presenting with a non-acute head injury or no head injury. During the same period, 1559 patients presenting to SECAmb with a head injury were non-conveyed, 125 (8.01%) of these patients were excluded, the leading reasons for exclusion were no evidence of a head injury or the patient was conveyed to hospital (the patient record had been miscoded), full details of patient flow can be found in Fig. 1. As these data were collected during the first year of the COVID-19 pandemic, month-on-month conveyance rates were reviewed. More patients were conveyed than non-conveyed each month except for April, where non-conveyance was higher (Fig. 2). Over half of the patients in the dataset were conveyed to the ED (2111/3545; 59.54%), and of those conveyed, most received a head CT scan (1600/2111; 75.79%), with 162 (10.12%) patients found to have a tICH on their scan. Whilst most patients with a tICH were referred to neurosurgery (148/162; 91.35%), eight (5.40%) patients were accepted.

### Conveyed patients

There was an equal split of genders between tICH and non-tICH groups (*p* = 0.9), and there was no significant difference in age between tICH and non-tICH groups (82 [75–88] vs 83 [74–89], respectively, *p* = 0.6). Falls from standing height were the most common cause of injury (1097, 90%). There was no difference between groups in visible head injury (123/162, 76% vs 1485/1949, 76%, *p* = 0.9). But, over half of the tICH patients presented with a head injury symptom (87/162, 54% vs 715/1949, 37%; p < 0.001). An abnormal GCS was found in a higher proportion of tICH patients (43/162, 27% vs 200/1949, 10%; *p* < 0.001). However, 62/162 (38%) patients in the tICH group presented with neither a head injury symptom nor abnormal GCS. An SDH was the most common type of brain injury (97/162, 59%). Most patients conveyed to the ED were discharged (1421/2111, 68%), and patients who were admitted into the hospital from the non-tICH group were admitted into acute medicine (207/1949, 11%). A third of patients found to have tICH were admitted under general surgery (55/162, 34%). A full comparison can be found in “Appendix B”.

### Predictors of an intracranial haemorrhage

**Fig. 3:**
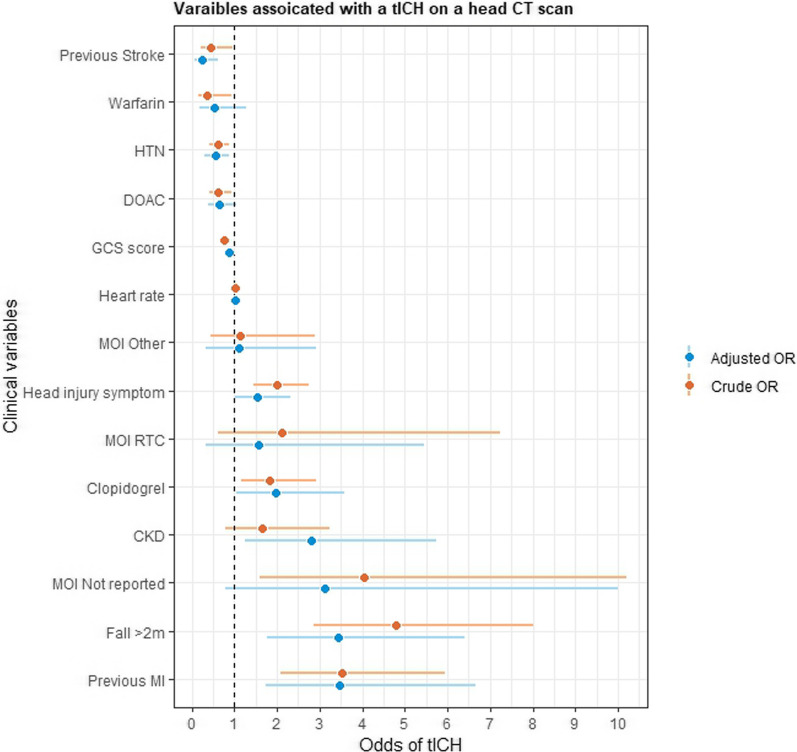
Crude and adjusted odds ratios of clinical variables associated with a traumatic intracranial haemorrhage (tICH). CKD, chronic kidney disease; DOAC, direct oral anticoagulant; GCS; Glasgow coma scale, HTN, hypertension; MI, myocardial infarction; RTC, road traffic collision

Univariate analysis revealed that patients taking Clopidogrel were at higher odds of an intracranial haemorrhage (OR 1.83; 95% CI 1.13–2.86), whereas patients on Warfarin or a direct oral anticoagulant (DOAC) were at greater odds of not having an intracranial haemorrhage (OR 0.37, 95% CI 0.13–0.83; OR 0.62, 95% CI 0.41–0.91, respectively). Patients with a previous MI were at greater odds of a tICH (OR 3.52, 95% CI 2.04–5.52), as were those patients with a head injury symptom (OR 2.00, 95% CI 1.45–2.77). Multivariable regression analysis produced a model with the most important clinical variables associated with an intracranial haemorrhage following stepwise variable selection (Fig. 3). Clopidogrel, when adjusted for other variables, remained associated with tICH (aOR 1.98, 95% CI 1.04–3.59), as was a previous history of CKD (aOR 2.8, 95% CI 1.25–5.75), a previous MI (aOR 3.48, 95% CI 1.73–6.65) and the presence of a neurological symptom (aOR 1.54, 95% CI 1.02–2.32). The higher the patient's GCS score, the lower the odds of an intracranial haemorrhage (aOR 0.87, 95% CI 0.75–1.00). Falls from > 2m remained the strongest predictor of an intracranial haemorrhage (aOR 3.45, 95% CI 1.78–6.40).

### Non-conveyed patients

The median age of non-conveyed patients was older than conveyed patients (84 years [76–90] vs 83 [75–89], *p* < 0.001). Patients who were non-conveyed were more likely to be females than males overall (2021 [57%] vs 1524 [43%], respectively, *p* = 0.021). An ambulance paramedic was in attendance in most cases (2568, 72%). A full comparison can be found in “Appendix C”.

### Predictors of conveyance

**Fig. 4:**
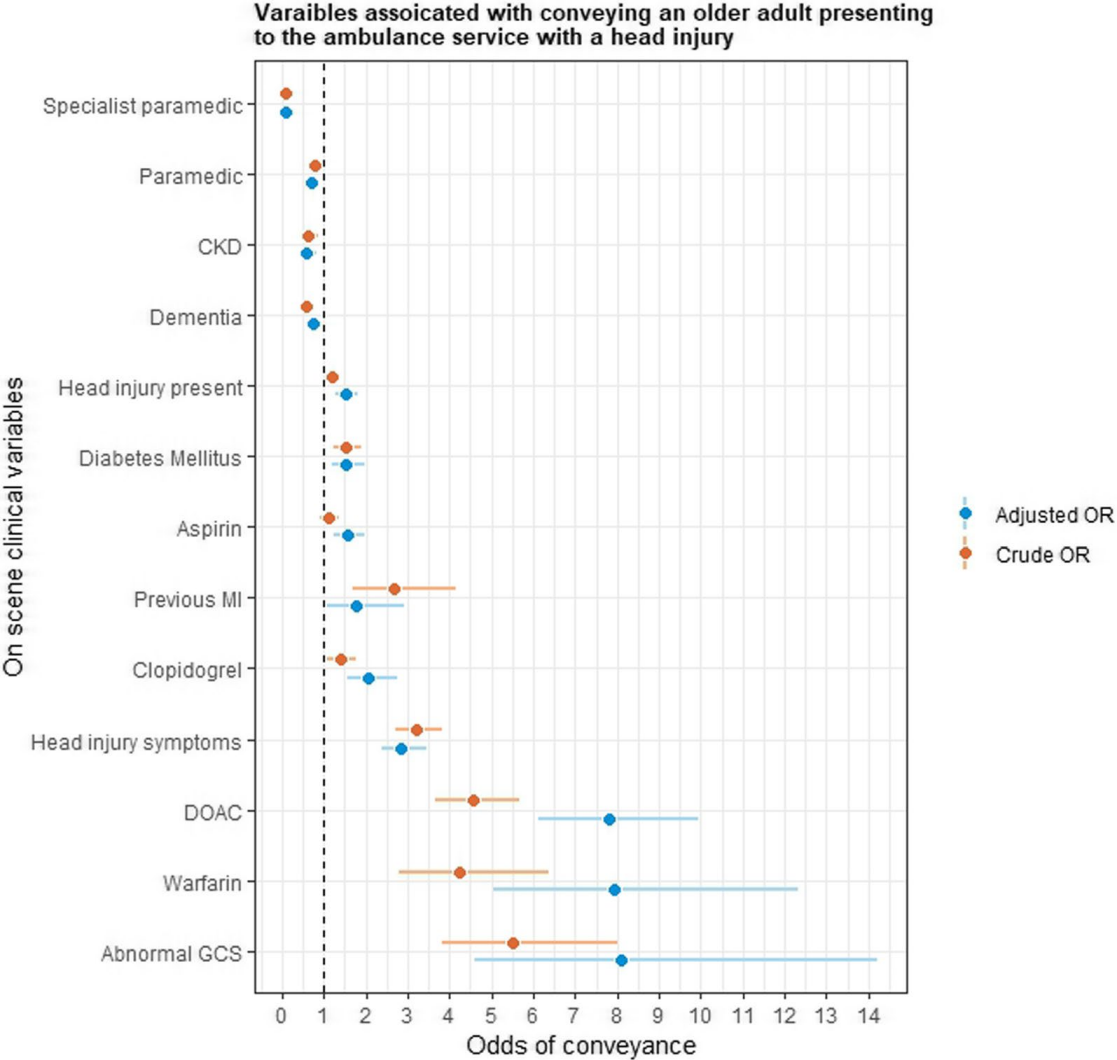
Crude and adjusted odds ratios of variables associated with conveyance to the emergency department. CKD, chronic kidney disease; DOAC, direct oral anticoagulant; GCS, Glasgow coma scale; MI, myocardial infarction

There was an association between the type of clinician present and whether a patient was conveyed; when compared to non-paramedic crews, the odds of conveyance decreased with a paramedic crew and more so if a specialist paramedic was present (OR 0.80, 95% CI 0.67–0.97; OR 0.10 95% CI 0.07–0.13). Patients taking antiplatelet or anticoagulant medications were at greater odds of being conveyed to the ED, as were the presence of a visible head injury, symptoms of a head injury or an abnormal GCS, higher heart rates and blood pressure were also associated with conveyance. However, lower alert levels, measured by the AcVPU scale, were more likely to be associated with non-conveyance. Equally, patients with dementia or CKD had greater odds of being non-conveyed. When odds ratios were adjusted for other variables, antiplatelet and anticoagulant medications remained strongly associated with conveyance, as did an abnormal GCS and the presence of a head injury. The clinical variables found to be significant in predicting conveyance are presented in Fig. 4, and full results can be found in “Appendix D”.

## Discussion

This is the first study to consider the clinical variables available to ambulance clinicians on scene and their association with tICH and conveyance to the hospital in older adults presenting with a head injury. Of the 3454 patients who presented to one regional ambulance service in England in 2020, 2111 (59.54%) patients were conveyed to the ED by SECAmb crews, of which only 162 (10.12%) were found to have a tICH on their head CT scan, consistent with the wider literature [16–21]. However, in their systematic review of older adults who presented to the ED with a fall, de Wit, Merali, et al. [19] reported a pooled incidence of tICH in older adults was 5.2% (95% CI 3.2–8.2%), the lower incidence rate compared to the findings in this study could be accounted for the inclusion of all types of MOI. Nevertheless, it indicates that in older adults presenting with a head injury, only a small proportion will have a tICH. Furthermore, several clinical variables were identified as being associated with a tICH. Clopidogrel and not Warfarin or DOAC was associated with a tICH, as was a previous history of an MI or CKD. In addition, head injury symptoms but not an abnormal GCS or a visible head injury were associated with a tICH.

Older adults with a head injury presenting to acute healthcare services such as the ambulance service or ED are a complex patient population to triage. For example, Kehoe et al. [5] demonstrated that older patients with an abbreviated injury score (AIS) head of four to six were likely to have a higher GCS score than young adults with a similar AIS head score and proposed that the GCS score may not be suitable in assessing the severity of a head injury in older adults. The findings presented in this study support Kehoe et al. [5], as an abnormal GCS score or the patient's actual GCS score (which was not included in the final model) was not associated with a tICH. However, the presence of head injury symptoms and not age was associated with a tICH; this is an important finding in that current head CT guidelines have identified adults over the age of 65 as a high-risk group and led clinicians to believe older age is a risk factor in itself [13]. Head CT guidelines are supported by several studies that recruited symptomatic head-injured patients in the ED, and as such, older adults represented a small proportion of the study population [16–22]. The critical factor here is that these patients were considered high-risk because of their age and were symptomatic. The findings from our study would discourage the use of chronological age in non-symptomatic head-injured patients. Furthermore, ambulance clinicians such as paramedics and specialist paramedics may make more pragmatic conveyance decisions due to an association with non-conveyance in older age (aOR 0.98, 95% CI 0.97–0.99).

The authors acknowledge that there was a small group of patients who were found to have a tICH but presented with no symptoms or an abnormal GCS (62/162; 38.27%). However, the absence of patient outcome data makes interpreting these findings difficult. Nevertheless, this is something that is rarely reported in the literature. When determining which older adults who were prescribed Warfarin or Clopidogrel presented with a head injury should be prioritised for a head CT scan, Nishijima et al. [23] noted that 12% of patients did not present with clinical symptoms of a head injury, and 75% did not have a history of loss of consciousness or amnesia. While a direct comparison between our findings and that of Nishijima et al. [23] is difficult, it highlights that a group of older adults may suffer a tICH without symptoms. Therefore, it is plausible that a group of patients in the non-conveyed cohort could have had a tICH. However, we would argue that given most patients who were conveyed to the ED did not have a tICH, with clinical support from senior colleagues, ambulance clinicians could make more pragmatic decisions on whether an asymptomatic older adult should be conveyed to the ED, especially those patients on anticoagulant medications. This could include consideration of other care pathways which avoid ED admissions [24, 25]. Further work is required to better understand the risk of non-conveying older adults with a head injury.

Ambulance clinical practice guidelines in the UK (JRCALC) state that patients who have hit their head and are taking anticoagulant medication (Warfarin or DOACs) should be conveyed to the ED, regardless of symptoms. These medications were found to be, unsurprisingly, associated with conveyance to the ED. However, only Clopidogrel was associated with increased odds of a tICH in patients conveyed to the ED. This is an important consideration, as Clopidogrel is an P2Y_12_ inhibitor reducing platelet aggregation, an important process in plugging ruptures in the vascular wall. Considerable attention has been paid to antiplatelet and anticoagulant medications in the literature where head injuries are concerned [26–31]. In their systematic review and meta-analysis, Santing et al. [30] reported that the risk of tICH in older adults taking DOAC was lower than those taking Warfarin and was comparable in patients taking antiplatelet medication. However, much of the evidence from systematic reviews was built from retrospective observational data [29, 30], the results of which may have influenced guidelines to be cautious. Our study's findings would support that current guideline recommendations are useful in symptomatic patients, but arguably, ambulance clinicians could take a more pragmatic approach to asymptomatic patients. While the findings in this study were based on retrospective data, they provide further evidence there is less risk of tICH from anticoagulant medication. This may help inform ambulance clinicians that asymptomatic head-injured patients may not require conveyance to the ED despite being prescribed anticoagulant medicines.

There is limited research on older adults presenting to the ambulance service with head injuries. Nicholson et al. [9] interviewed paramedics (n = 10) on the factors associated with conveyance decision-making; paramedics remarked that the lack of appropriate alternative care pathways, wound management and stringent guidelines make it challenging to non-convey older adults with a head injury. Our study found that older age and cognitive impairment were associated with non-conveyance, perhaps a surrogate marker for frailer older adults where ED services may not be appropriate for these patients. This may also suggest that ambulance clinicians make pragmatic decisions about the needs of the patients. Nevertheless, the association with conveyance mirrored guideline recommendations. While a visible head injury was associated with conveyance, it was not predictive of a tICH, perhaps, amongst other factors, reflecting the lack of available wound care skill to non-extended scope of practice clinicians. This is supported by the strong association of non-conveyance by specialist paramedics (OR 0.10, 95% CI 0.07–0.13) compared to non-paramedic crews, and that an extended scope of knowledge, clinical skill and decision-making maybe beneficial to this patient population. Interestingly, 75% of non-tICH patients were discharged from the ED. While this should be interpreted with caution, there may be a place for ED treatment and review which is beyond the scope of ambulance clinicians. Equally, the literature suggests paramedics would rather access alternative care pathways for their older adult head-injured patients, but these services are unavailable [9].

Comorbidities and their significance in conveyance decision-making and the risk of tICH should not be overlooked. The underlying pathophysiological processes caused by comorbidities may put a patient at greater risk of a tICH following head trauma. For example, in their observational study designed to identify predictors of TBI in older adults attending the ED following a head injury, de Wit et al. [10] found that patients with a history of CKD were at greater risk of tICH. Although similar findings were presented in this study, previous MI or CKD were also associated with a tICH. While the significance of these findings remains to be understood, evidence in stroke care suggests that raised albumin levels from CKD contribute to endothelial dysfunction and vascular damage and increases the chance of a haemorrhagic stroke [32]. Therefore, it is plausible that certain comorbidities place patients at greater risk of intracranial bleeding through progressive weakening of vascular structures.

## Limitations

Several limitations to this study should be acknowledged; the retrospective case–control study is susceptible to selection bias. By selecting patients based on their ED disposition and not the ePCR coding, it is possible that while a head injury may be present, it was not the primary reason the patient was conveyed to the hospital; for example, a medical episode may have caused a fall and the patient required to follow up investigations. However, using the ED system to search for patients allows for greater accuracy in findings for all patients with a head injury. Using an intracranial haemorrhage as an outcome measure is pragmatic but may not be correlated to neurosurgical intervention. Bleed sizes were not routinely recorded as were reasons for not accepting a patient under neurosurgery. Where a reason was recorded, it stated the patient was for conservative management, but no context to the decision was provided. There was a significant amount of missing data in the ePCR; assumptions were made for missing data; for example, past medical history and prescribed medications only required clinicians to document when they were present. Therefore, a patient was considered not to have a condition or medication if this was absent; however, clinicians can indicate whether a patient has no past medical conditions by ticking a box; if these fields were left blank and the appropriate box not ticked, then these data were considered missing. A predefined list of known injuries and symptoms associated with a head injury were searched in the clinical text to determine the presence or absence of symptoms. The absence of an injury or symptom was treated as missing on the assumption that clinical record keeping should document whether they were present at the time of assessment. Composite variables were created to ensure data were not needlessly lost but may not represent clinical practice. It is not possible to know what a head CT scan would have shown in patients who did not receive one in the ED or in those patients who were non-conveyed. Whilst a head CT scan is a pragmatic outcome measure it does not relate to a requirement for intervention and conservative management would be an appropriate course of management for a patient. The clinical variables included in the analysis were only available in the dataset, and other unmeasured variables may influence the findings of these results. For example, clinical frailty scores have been gaining importance in older adult trauma research; however, these measures were missing in a significant proportion of cases and may influence whether a patient is at risk of a TBI or impact conveyance decision-making. Finally, the data collection period occurred during the start of the COVID-19 pandemic, and conveyance behaviour only changes during the month of April (Fig. 2) it is unclear whether the demands placed on healthcare systems because of COVID-19 influenced in-hospital clinical practice in the case of head injuries in older adults.

## Conclusion

The findings from this study present a unique insight into older adults presenting to the ambulance service with a head injury. Over half of these patients are conveyed to the ED, but only a small proportion will have a tICH. Most patients that do not have a tICH are discharged from the ED. Therefore, alternative care pathways may be more appropriate for some of these patients, and further support may be required to allow ambulance clinicians to access these pathways. Equally, these findings may support the re-evaluation of ambulance clinical guidelines. Finally, a group of patients may have a tICH but present without symptoms; it is unclear what the implications of this are for older adults as their TBI would likely be missed. Further work is required to determine which head-injured patients should be conveyed to the ED.

## Appendix A: Example of SOMED Codes

**Table:**

		Head injury patients who received a head CT scan
	*11.1 Arrival mode*	
	*ECDS_UniqueID*	
	2018310000	Arrival by emergency road ambulance (finding)
OR	2018350000	Arrival by emergency road ambulance with medical escort (finding)
OR	2018510000	Arrival by helicopter Air Ambulance (finding)
AND	*11.4 Chief Complaint*	
	1161111000	Head injury
OR	1161131000	Facial injury
AND	*20.1 Diagnosis*	
	121111111000	Open wound: scalp
OR	121121111000	Abrasion: head
OR	121131111000	Bruise: head
OR	121161111000	Crush injury: head
OR	121171111000	Degloving injury: head
OR	122511111000	Head injury: no LOC
OR	122511131000	Minor head injury: LOC less than 30 s
OR	122511151000	Minor head injury: LOC more than 30 s
OR	122511811000	Moderate head injury (GCS less than 13)
OR	122511851000	Severe head injury: GCS less than 9
AND	*20.2 Diagnosis Qualifier*	
	2018110000	Suspected diagnosis
OR	2018210000	Confirmed diagnosis
AND	*21.1 Investigations*	
	1171410000	Computerised axial tomography (procedure)
		*Head injury patients who did not receive a head CT scan*
	*11.1 Arrival mode*	
	*ECDS_UniqueID*	
	2018310000	Arrival by emergency road ambulance (finding)
OR	2018350000	Arrival by emergency road ambulance with medical escort (finding)
OR	2018510000	Arrival by helicopter Air Ambulance (finding)
AND	*11.4 Chief Complaint*	
	1161111000	Head injury
OR	1161131000	Facial injury
AND	*20.1 Diagnosis*	
	121111111000	Open wound: scalp
OR	121121111000	Abrasion: head
OR	121131111000	Bruise: head
OR	121161111000	Crush injury: head
OR	121171111000	Degloving injury: head
OR	122511111000	Head injury: no LOC(Traumatic brain injury with no loss of consciousness)
OR	122511131000	Minor head injury: LOC less than 30 s (Traumatic brain injury with brief loss of consciousness)
OR	122511151000	Minor head injury: LOC more than 30 s (Traumatic brain injury with moderate loss of consciousness)
OR	122511811000	Moderate head injury (GCS less than 13)
OR	122511851000	Severe head injury: GCS less than 9
AND	*20.2 Diagnosis Qualifier*	
	2018110000	Suspected diagnosis
OR	2018210000	Confirmed diagnosis
	*21.1 Investigations*	
NOT	1171410000	Computerised axial tomography (procedure)

## Appendix B: Comparison of no tICH and tICH on head CT

See Table 1.

**Table 1:** Comparision of patients found to have a tICH on head CT compared to patients with no tICH found on head CT

	Overall, N = 2111	No tICH on CT, N = 1949	tICH on CT, N = 162	*p*-value
*Gender*				0.9
Female	1170 (55%)	1081 (55%)	89 (55%)	
Male	941 (44%)	868 (45%)	73 (45%)	
Age	83.00 (75, 89)	83.00 (74, 89)	82.00 (75, 88)	0.6
*Ethnicity*				0.038
Not stated	319 (17%)	287 (17%)	32 (24%)	
Other ethnic backgrounds	13 (0.7%)	12 (0.7%)	1 (0.7%)	
White British	1499 (81%)	1402 (81%)	97 (72%)	
White other	26 (1.4%)	22 (1.3%)	4 (3.0%)	
Unknown	255	227	28	
*Priority*				< 0.001
Category 1	50 (2.4%)	37 (1.9%)	13 (8.0%)	
Category 2	924 (44%)	843 (43%)	81 (50%)	
Category 3	1108 (53%)	1042 (54%)	66 (41%)	
Category 4	25 (1.2%)	23 (1.2%)	2 (1.2%)	
Unknown	4	4	0	
*Mechanism of injury*				< 0.001
Fall	1907 (90%)	1781 (91%)	126 (78%)	
Fall > 2 m	87 (4.1%)	65 (3.3%)	22 (14%)	
Not reported	27 (1.3%)	21 (1.1%)	6 (3.7)	
Other	67 (3.2%)	62 (3.2%)	5 (3.1%)	
RTC	23 (1.1%)	20 (1.0%)	3 (1.9%)	
*Preinjury medications*				
Aspirin	297 (15%)	267 (15%)	30 (20%)	0.09
Clopidogrel	193 (9.1%)	169 (8.7%)	24 (15%)	0.009
DOAC	585 (28%)	553 (28%)	32 (20%)	0.018
Warfarin	158 (7.5%)	153 (7.9%)	5 (3.1%)	0.027
*Preinjury comorbidities*				
Alcohol dependence	60 (2.8%)	55 (2.8%)	5 (3.1%)	0.8
Alzheimer's disease	111 (5.3%)	105 (5.4%)	6 (3.1%)	0.4
Atrial fibrillation	459 (22%)	432 (22%)	27 (17%)	0.1
CKD	85 (4.0%)	75 (3.8%)	10 (6.3%)	0.15
COPD	173 (8.2%)	156 (8.0%)	17 (10%)	0.3
Dementia	401 (19%)	369 (19%)	32 (20%)	0.8
Diabetes Mellites	302 (14%)	282 (15%)	20 (12%)	0.5
Heart failure	96 (4.5%)	91 (4.7%)	5 (3.1%)	0.4
Hypertension	706 (33%)	667 (34%)	39 (24%)	0.009
Ischemic heart disease	49 (2.3%)	46 (2.4%)	3 (1.9%)	> 0.9
Myocardial infarction	95 (4.5%)	75 (3.8%)	20 (12%)	< 0.001
Stroke	189 (9.0%)	182 (9.3%)	7 (4.3%)	0.032
*Clinical assessment*				
Abnormal GCS	243 (12%)	200 (10%)	43 (27%)	< 0.001
Focal neurological deficit	802 (38%)	715 (37%)	87 (54%)	< 0.001
Head injury present	1608 (76%)	1485 (76%)	123 (76%)	< 0.9
*Hospital Admission*				
Acute Assessment Unit	104 (4.9%)	84 (4.3%)	20 (12%)	
Acute Medicine	234 (11%)	207 (11%)	27 (17%)	
Cardiology	1 (< 0.1%)	1 (< 0.1%)	0 (0%)	
Discharged	1421 (68%)	1411 (73%)	6 (3.8%)	
General Medicine	89 (4.2%)	77 (4.0%)	12 (7.5%)	
General Surgery	67 (3.2%)	12 (0.6%)	55 (34%)	
Geriatric Medicine	90 (4.3%)	81 (4.2%)	9 (5.6%)	
Intensive Care	10 (0.5%)	3 (0.2%)	7 (4.4%)	
Neurosurgery	4 (0.2%)	3 (0.2%)	1 (0.6%)	
Orthopaedic	19 (0.9%)	14 (0.7%)	5 (3.1%)	
Other	21 (1.0%)	19 (1.0%)	2 (1.3%)	
Transfer—Neuro	5 (0.2%)	0 (0%)	5 (3.1%)	
Transfer—Other	15 (0.7%)	9 (0.5%)	6 (3.8%)	
Trauma and Orthopaedics	25 (1.2%)	20 (1.0%)	5 (3.1%)	
Unknown	6	4	2	

## Appendix C

See Table 2.

**Table 2:** Comparison of patients conveyed and non-conveyed by the ambulance service

Variable	Overall, N = 3545	Non-conveyed, N = 1434	Conveyed, N = 2111	*p*-value
*Gender*				0.021
Female	2021 (57%)	851 (59%)	1170 (55%)	
Male	1524 (43%)	583 (41%)	941 (45%)	
Age	83 (75, 89)	84 (76, 90)	83 (75, 89)	< 0.001
*Ethnicity*				0.012
Not stated	579 (19%)	260 (21%)	319 (17%)	
Other ethnic backgrounds	28 (0.9%)	15 (1.2%)	13 (0.7%)	
White British	2430 (79%)	931 (76%)	1499 (81%)	
White other	41 (1.3%)	15 (1.2%)	26 (1.4%)	
Unknown	467	213	254	
*Registrant present*				< 0.001
Non-registered	647 (18%)	208 (15%)	439 (21%)	
Paramedic	2568 (72%)	952 (66%)	1616 (77%)	
Specialist Paramedic	330 (9.3%)	274 (19%)	56 (2.7%)	
*Priority*				< 0.001
Category 1	62 (1.8%)	12 (0.8%)	50 (2.4%)	
Category 2	1279 (36%)	355 (25%)	924 (44%)	
Category 3	2135 (60%)	1027 (72%)	1108 (53%)	
Category 4	61 (1.7%)	36 (2.5%)	25 (1.2%)	
Unknown	8	4	4	
*MOI*				< 0.001
Fall	3252 (92%)	1345 (94%)	1907 (90%)	
Fall > 2m	91 (2.6%)	4 (0.3%)	87 (4.1%)	
Not reported	45 (1.3%)	18 (1.3%)	27 (1.3%)	
Other	122 (3.4%)	55 (3.8%)	67 (3.2%)	
RTC	35 (1.0%)	12 (0.8%)	23 (1.1%)	
*Preinjury medications*				
Aspirin	479 (14%)	182 (13%)	297 (14%)	0.2
Clopidogrel	290 (8.2%)	97 (6.8%)	193 (9.1%)	0.011
DOAC	696 (20%)	111 (7.7%)	585 (28%)	< 0.001
Warfarin	185 (5.2%)	27 (1.9%)	158 (7.5%)	< 0.001
*Preinjury comorbidities*				
Alcohol dependence	100 (2.8%)	40 (2.8%)	60 (2.8%)	> 0.9
Alzheimer's disease	214 (6.0%)	103 (7.2%)	111 (5.3%)	0.018
Atrial fibrillation	630 (18%)	171 (12%)	459 (22%)	< 0.001
CKD	173 (4.9%)	88 (6.1%)	85 (4.0%)	0.004
COPD	251 (7.1%)	78 (5.4%)	173 (8.2%)	0.002
Dementia	814 (23%)	413 (29%)	401 (19%)	< 0.001
Diabetes mellites	442 (12%)	140 (9.8%)	302 (14%)	< 0.001
Heart failure	143 (4.0%)	47 (3.3%)	96 (4.5%)	0.059
Hypertension	1140 (32%)	434 (30%)	706 (33%)	0.047
Ischemic Heart disease	74 (2.1%)	25 (1.7%)	49 (2.3%)	0.2
Myocardial infarction	120 (3.4%)	25 (1.7%)	95 (4.5%)	< 0.001
Stroke	281 (7.9%)	92 (6.4%)	189 (9.0%)	0.006
*Clinical assessment*				
Focal neurological deficit	1031 (29%)	229 (16%)	802 (38%)	< 0.001
Visible head injury	2654 (75%)	1046 (73%)	1608 (76%)	0.030
Abnormal GCS score	276 (7.8%)	33 (2.3%)	243 (12%)	< 0.001
Respiratory rate (br/pm)	18.0 (16.0, 18.0)	17.0 (16.0, 18.0)	18.0 (16.0, 18.5)	< 0.001
Sp02 (%)	97.00 (96.00, 98.00)	97.00 (96.00, 98.00)	97.00 (96.00, 98.00)	0.044
Heart rate (bpm	78 (68, 88)	76 (68, 85)	79 (68, 89)	< 0.001
Systolic blood pressure	145 (130, 164)	140 (126, 156)	150 (131, 170)	< 0.001
Diastolic blood pressure	80 (70, 90)	78 (70, 85)	80 (70, 92)	< 0.001
GCS score	15.00 (14.00, 15.00)	15.00 (15.00, 15.00)	15.00 (14.00, 15.00)	0.2
*GCS components*				
Eye				0.047
Open	3394 (96%)	1362 (95%)	2032 (96%)	
Verbal	94 (2.7%)	43 (3.0%)	51 (2.4%)	
Pain	34 (1.0%)	21 (1.5%)	13 (0.6%)	
Closed	23 (0.6%)	8 (0.6%)	15 (0.7%)	
*Voice*				0.006
Normal	2643 (75%)	1082 (75%)	1561 (74%)	
Confused	775 (22%)	285 (20%)	490 (23%)	
Incomprehensible	45 (1.3%)	23 (1.6%)	22 (1.0%)	
Inappropriate	37 (1.0%)	17 (1.2%)	20 (0.9%)	
Unresponsive	45 (1.3%)	27 (1.9%)	18 (0.9%)	
*Motor*				> 0.9
Obeys	3442 (97%)	1394 (97%)	2048 (97%)	
Localises	57 (1.6%)	22 (1.5%)	35 (1.7%)	
Withdraws	33 (0.9%)	13 (0.9%)	20 (0.9%)	
Extensor	4 (0.1%)	2 (0.1%)	2 (< 0.1%)	
No response	9 (0.3%)	3 (0.2%)	6 (0.3%)	
*AcVPU*				< 0.001
Alert	3282 (93%)	1336 (93%)	1946 (92%)	
Confused	158 (4.5%)	40 (2.8%)	118 (5.6%)	
Verbal	41 (1.2%)	19 (1.3%)	22 (1.0%)	
Pain	35 (1.0%)	21 (1.5%)	14 (0.7%)	
Unresponsive	29 (0.8%)	18 (1.3%)	11 (0.5%)	

## Appendix D

See Table 3.

**Table 3:** Crude and adjusted odds ratios of clinical variables associated with a traumatic intracranial haemorrhage (tICH)

Predictor of tICH	Crude OR	95% CI	Adj. OR	95% CI
Clopidogrel	1.83	1.15–2.91	1.98	1.04–3.59
DOAC	0. 62	0.42–0.93	0.64	0.38–1.05
Warfarin	0.37	0.15–0.92	0.54	0.18–1.28
MI	3.52	2.09–5.93	3.48	1.73–6.65
CKD	1.64	0.8–3.25	2.80	1.25–5.75
HTN	0.61	0.42–0.88	0.57	0.31–0.87
Stroke	0.44	0.2–0.95	0.24	0.08–0.62
*Clinical observations*				
Heart rate	1.02	1.01–1.03	1.02	1.01–1.03
GCS score	0.75	0.68–0.83	0.87	0.75–1.00
Head injury symptom	2.00	1.45–2.76	1.54	1.02–2.32
Fall	Ref	–	Ref	–
Fall > 2m	4.78	2.86–8.02	3.45	1.78–6.40
Not reported	4.04	1.6–10.19	3.13	0.78–10.00
RTC	2.12	0.62–7.23	1.56	0.32–5.46
Other	1.14	0.45–2.89	1.12	0.32–2.91

CKD, chronic kidney disease; DOAC, direct oral anticoagulant; GCS; Glasgow coma scale, HTN, hypertension; MI, myocardial infarction; RTC, Road traffic collision

## Appendix E

See Table 4.

**Table 4:** Crude and adjusted odds ratios of variables associated with conveyance to the emergency department

Predictors of conveyance	Crude OR	95% CI	adj. OR	95% CI
Age	0.99	0.98–0.99	0.98	0.97–0.99
Warfarin	4.22	2.79–6.38	7.91	5.06–12.34
Aspirin	1.13	0.92–1.37	1.57	1.25–1.98
Clopidogrel	1.39	1.08–1.79	2.08	1.56–2.76
DOAC	4.57	3.68–5.67	7.82	6.14–9.96
Previous MI	2.66	1.7–4.15	1.76	1.07–2.92
Dementia	0.58	0.5–0.68	0.73	0.59–0.9
Diabetes Mellitus	1.54	1.25–1.91	1.54	1.2–1.97
CKD	0.64	0.47–0.87	0.57	0.4–0.83
Head injury symptoms	3.22	2.73–3.81	2.85	2.37–3.44
Head injury present	1.19	1.02–1.38	1.52	1.27–1.83
Abnormal GCS	5.52	3.81–8.00	8.09	4.61–14.21
Respiratory rate	1.10	1.07–1.12	1.06	1.03–1.09
Heart rate	1.01	1.01–1.02	1.01	1.01–1.02
Blood pressure—Systolic	1.01	1.01–1.02	1.01	1.01–1.02
*GCS—Verbal*				
Normal	Ref		Ref	
Confused	1.19	1.01–1.41	1.27	0.99–1.61
Incomprehensible	0.66	0.37–1.20	0.50	0.21–1.2
Inappropriate	0.82	0.43–1.56	0.61	0.26–1.44
Unresponsive	0.46	0.25–0.84	0.57	0.24–1.35
*AcVPU*				
Alert	Ref		Ref	
Confused	2.03	1.41–2.92	1.17	0.72–1.89
Verbal	0.79	0.43–1.47	0.34	0.14–0.82
Pain	0.46	0.23–0.90	0.11	0.04–0.29
Unresponsive	0.42	0.2–0.89	0.39	0.14–1.09
*Clinician on scene*				
Non-registered	Ref		Ref	
Paramedic	0.80	0.67–0.97	0.70	0.56–0.88
Specialist paramedic	0.10	0.07–0.13	0.07	0.05–0.12

CKD, chronic kidney disease; DOAC, direct oral anticoagulant; GCS, Glasgow coma scale; MI, myocardial infarction
